# Electrospinning: Application and Prospects for Urologic Tissue Engineering

**DOI:** 10.3389/fbioe.2020.579925

**Published:** 2020-10-07

**Authors:** Masoud Zamani, Nasser Shakhssalim, Seeram Ramakrishna, Mohammad Naji

**Affiliations:** ^1^Department of Chemical and Biological Engineering, University at Buffalo, State University of New York, Amherst, NY, United States; ^2^Urology and Nephrology Research Center, Shahid Beheshti University of Medical Sciences, Tehran, Iran; ^3^Department of Mechanical Engineering, National University of Singapore, Singapore, Singapore

**Keywords:** biopolymers, regeneration, scaffold, urinary tract, urethra, ureter, urinary bladder, electrospinning

## Abstract

Functional disorders and injuries of urinary bladder, urethra, and ureter may necessitate the application of urologic reconstructive surgeries to recover normal urine passage, prevent progressive damages of these organs and upstream structures, and improve the quality of life of patients. Reconstructive surgeries are generally very invasive procedures that utilize autologous tissues. In addition to imperfect functional outcomes, these procedures are associated with significant complications owing to long-term contact of urine with unspecific tissues, donor site morbidity, and lack of sufficient tissue for vast reconstructions. Thanks to the extensive advancements in tissue engineering strategies, reconstruction of the diseased urologic organs through tissue engineering have provided promising vistas during the last two decades. Several biomaterials and fabrication methods have been utilized for reconstruction of the urinary tract in animal models and human subjects; however, limited success has been reported, which inspires the application of new methods and biomaterials. Electrospinning is the primary method for the production of nanofibers from a broad array of natural and synthetic biomaterials. The biomimetic structure of electrospun scaffolds provides an ECM-like matrix that can modulate cells’ function. In addition, electrospinning is a versatile technique for the incorporation of drugs, biomolecules, and living cells into the constructed scaffolds. This method can also be integrated with other fabrication procedures to achieve hybrid smart constructs with improved performance. Herein, we reviewed the application and outcomes of electrospun scaffolds in tissue engineering of bladder, urethra, and ureter. First, we presented the current status of tissue engineering in each organ, then reviewed electrospun scaffolds from the simplest to the most intricate designs, and summarized the outcomes of preclinical (animal) studies in this area.

## Introduction

The normal function of different organs in the urinary tract could be compromised in several clinical conditions (Bauer, 2008; Delacroix and Winters, 2010). Functional disorders of bladder, urethra, and ureter may be ensued by secondary sequels in other organs, especially the kidney, and finally, end in immense and irreparable consequences in the quality of life of patients. Therefore, the malfunction of these organs necessities the urologists to intervene through reconstructive surgeries to recover the lost functions. The current surgical treatments are generally based on the use of autologous patients’ tissues, which unsurprisingly are associated with donor and recipient site morbidity (Veeratterapillay et al., 2013; Lumen et al., 2016). These reconstructive procedures are very invasive and cannot be carried out for all the patients with diverse clinical situations readily, especially for those who extensively damaged that lack sufficient autologous tissue (Browne and Vanni, 2017). In addition, because of imperfect functional outcomes and complicating consequences of current standard reconstructive surgical methods, there have been continuous efforts to discover new therapeutic strategies.

During the last two decades, tissue engineering through the integration of cell-based methods, material and engineering sciences, and biomimetic approaches has developed promising outlooks for finding novel and innovative strategies for recovering of compromised functions of diseased organs. To this end, numerous works have been conducted to study new biomaterials and fabrication techniques. Today, tissue engineering is still in its infancy, but in the near future, it will definitely have an indisputable role as a regenerative modality for patients.

**FIGURE 1:**
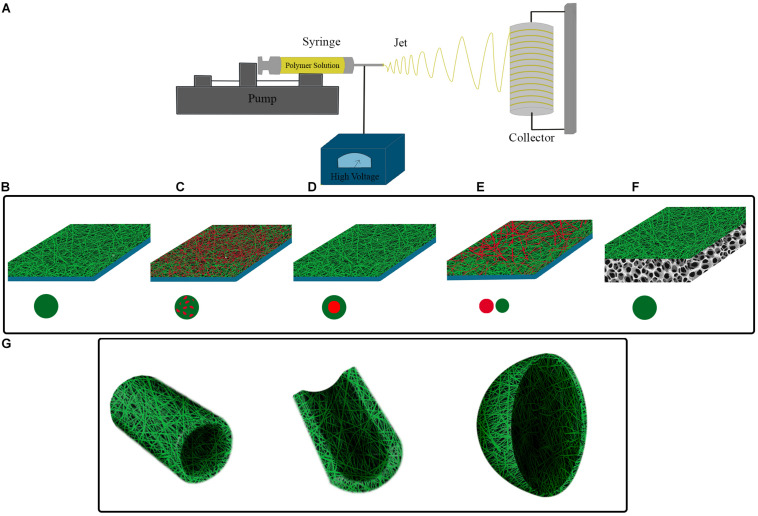
**(A)** Electrospinning set-up. Polymer solution is electrospun in high voltage field onto a collecting surface. Different electrospinning parameters such as polymer solution, voltage intensity, and syringe tip to collecting surface distance are adjustable for construction of electrospun scaffolds with intended features. Single or multiple material (polymer) can be utilized for fabrication of different electrospinning scaffolds. **(B)** A simple electrospun scaffold contains a single polymer in fibers composition. Composite electrospun scaffolds have different materials in their structure, this can be obtained by **(C)** using a mixture of polymers in a single syringe, **(D)** utilizing coaxial strategy, and **(E)** simultaneous electrospinning of polymers from distinct syringes. **(F)** Hybrid electrospun scaffolds can be constructed by electrospinning of polymers onto the scaffolds, which have been prepared by other fabrication method. **(G)** Considering the shape of target tissue and injury location, nanofibers can be electrospun onto pre-fabricated molds with corresponding structure.

Based on biomimetic principles, a desirable tissue-engineering scaffold should not only have appropriate biocompatibility and bioactivity, but also should have physical architecture and mechanical properties of the natural extracellular matrix (ECM) of that specific tissue to efficiently support the cells colonization, differentiation, and tissue remodeling (Yao et al., 2016; Pazhanimala et al., 2019). To mimic the nanoscale structure of ECM, various scaffold production techniques can be employed, such as phase separation, self-assembly, fused deposition modeling, and electrospinning, among which the latter one holds the greatest promise due to several advantages (Bhattarai et al., 2018). Electrospinning is the most simple and efficient method to produce three dimensional scaffolds with controllable porosity and mechanical properties composed of very thin fibers with diameters ranging from tens of nanometers to micrometers covering the structural dimension of ECM of most native tissues (Figure 1A). Furthermore, it is so versatile that enables the use of a variety of natural and synthetic polymers and incorporation of drugs, biomolecules, and living cells for addressing different application requirements (Zhang et al., 2005; Bhattarai et al., 2018). Due to these advantages, electrospinning has been widely investigated for various tissue engineering applications and proved to have the potential for some clinical applications. Considering biomaterials field, several electrospun products have been commercially introduced to the market, such as coronary balloon expandable stent system, utilized polyurethane for higher of stent (Papyrus, Biotronik AG and Bioweb^TM^, Zeus Inc.), vascular access graft (AVflo^TM^, Nicast Ltd.), dural patch (ReDura^TM^, MEDPRIN Regenerative Medical Technologies Co., Ltd.), and synthetic bone (ReBOSSIS, ORTHOReBIRTH Co., Ltd.) (Liverani et al., 2017; Liu et al., 2020).

This review article gives a brief overview of the anatomy and function of the lower urinary tract and the current state of achievements in the tissue engineering of urologic tissues. Then critically evaluates the advantages of different biopolymers and design strategies for the development of electrospun-based scaffolds for the engineering of these tissues.

## Search Strategy and Study Design

In the current study, we reviewed the studies, which utilized electrospun scaffolds for tissue reconstruction of bladder, urethra, and ureter. Both *in vitro* and preclinical works were included in this study. Specific search filters for bladder, urethra, ureter, tissue engineering, and electrospinning were customized. The search filter for each organ was combined with tissue engineering, and electrospinning filters, and the search was carried out in PubMed. Scopus and Google scholar were also searched for pertinent studies that have not been found by PubMed search engine. The search results in each field (organ) were trimmed by removing studies not related to the specific organ, no tissue engineering strategy applied, not being original, without electrospinning fabrication method and, lack of full text (Supplementary Material). Flowcharts of search and screening procedure for studies concerning bladder, urethra, and ureter were depicted in Supplementary Figures 1–3, respectively. For each organ, a brief description of tissue engineering status and prominent works (including human trials) was reviewed at first. Next, the electrospun scaffolds were classified based on their components into simple, composite, and hybrid; and reviewed, respectively.

## Bladder Tissue Engineering

**FIGURE 2:**
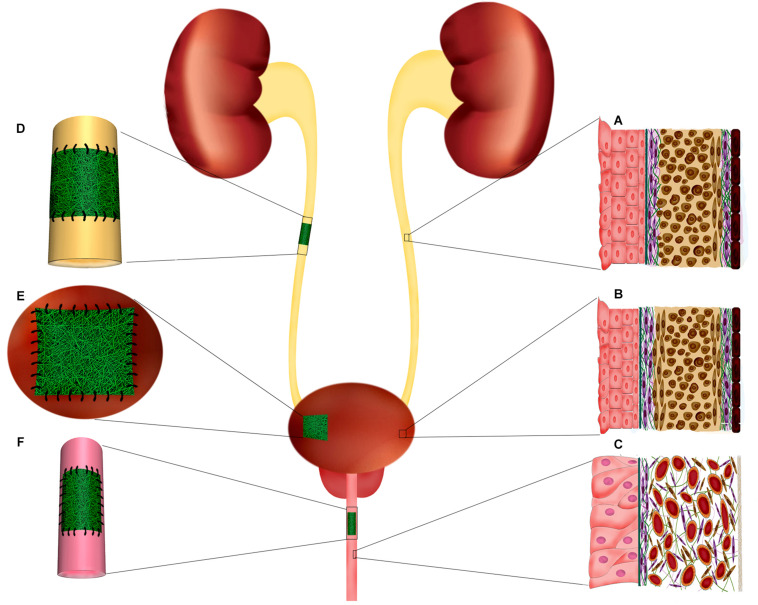
Ureter, urinary bladder, and penile urethra histological structure. Implantation of electrospun scaffolds in the urinary tract organs for reconstructive purposes through tissue engineering. **(A)** Schematic organization of ureter layers. The innermost layer, adjacent to the lumen, composed of specialized epithelium, urothelium, which protects the deeper layers from urine. The muscular layer of ureter is mostly composed of a longitudinally oriented inner layer and a circular outer layer. **(B)** Bladder histological organization. The inner transitional epithelium, urothelium, comprises 4–5 layers of highly specialized cells including umbrella cells at urine interface and basal cell adjacent to the basal lamina and submucosa. Bladder wall is mainly formed by a thick muscular layer (60–70% of normal bladder wall thickness) which is the major contributor of resilient nature of bladder wall. The bladder muscular layer is structurally organized as two outer longitudinal muscular layers intervened by circumferentially oriented muscular cells. **(C)** In the penile urethra the epithelium is mostly composed of pseudostratified columnar epithelium. The submucosa contains many venous spaces encompassed in connective tissue. **(D)** In ureter injuries, where end-to-end anastomosis is no possible, a tubular electrospun construct can be implanted to fill the injury and support the regeneration of ureter layers. **(E)** Augmentation cystoplasty is required for patients with diseased bladder that lost their functionality for being compliant and low-pressure reservoir. Electrospun scaffolds can provide a supportive matrix for regeneration of bladder tissue in partial augmentation cystoplasty. **(F)** Affected urethral wall with stricture lesion can be replaced by an electrospun scaffold through onlay or inlay fashion.

Urinary bladder functions by providing a low-pressure reservoir for urine storage and periodic voluntary voiding. In the bladder wall, the specialized epithelial lining, urothelium, and the smooth muscle layer, detrusor muscle, are integral components that guarantee a compliant bladder with proper emptying capacity; thereby, protecting the upper urinary tract from damaging effects of high-pressure urine storage (Figure 2B). At the luminal surface of the bladder, the urothelium layer directly interfaces with the complex environment of urine. Thus, its proper function as a blood-urine barrier is critical for protecting deeper layers of the bladder from urine extravasation. Three specialized structures are responsible for the impermeability of the urothelium barrier: (1) asymmetric unit membranes in the apical membrane of superficial cells, umbrella cells, that mainly composed of uroplakin proteins, (2) occluding junctions between umbrella cells, and (3) glycocalyx of umbrella cells (Yamany et al., 2014). These features enable the urothelium to maintain the impermeable barrier even when the internal pressure increases by detrusor muscle contraction during voiding (Apodaca, 2004). The resilient nature of the bladder is primarily retained by the normal function of the detrusor muscle. This muscular layer encompasses about 60–70% of normal bladder wall thickness, in which three structural layers can be identified; the muscular cells in the outer and inner layers are oriented longitudinally, while in the middle layer the cells are circumferentially oriented. The detrusor muscle in orchestration with neural and vascular plexus of bladder provide a passive elastic reservoir for urine storage (Andersson and Arner, 2004; Ajalloueian et al., 2018). ECM is another critical player in the biomechanics of the bladder. The characteristic composition of ECM in different layers of bladder regulates the behavior of the cells and, so, the physiology of the whole organ (Nagatomi et al., 2008; Aitken and Bägli, 2009). It is a dynamic environment with a high turnover of constituent elements. In this regard, pathologic conditions may alter the composition of ECM to the extent that it affects the biomechanics of the bladder and, eventually, its normal function (Aitken and Bägli, 2009).

Several conditions in urology practice demand surgeons’ intervention to prevent progressive damage of the diseased organs, and restore the impaired function. Neurogenic bladder, inflammation, trauma, and iatrogenic injury may compromise the normal function of the bladder and lead to urine storage at high pressure, which can be associated with detrimental consequences for the kidney, and sphincter of the urethra (Biers et al., 2012). Hence, a progressive increase in bladder pressure has to be alleviated to prevent kidney failure and incontinency. Also, malignancies of the bladder usually require radical or partial removal of the organ, requiring urgent new structures for either storage or diversion of urine flow. Usually, patients’ own tissues are the first option for reconstruction purposes. Enterocystoplasty employs a segment of patients’ intestine for augmentation of the bladder capacity and improvement of its compliance, which can relief the kidneys and the sphincter from the high pressure of the bladder. However, as intestinal tissue is not naturally specialized for urine exposure, the long-term contact of an absorptive epithelium with urine leads to serious complications including metabolic disturbance, renal dysfunction, diverticularization, mucus production, stones formation, perforation of bladder, malignancy, and infection (Bertschy et al., 2000; Biers et al., 2012; Veeratterapillay et al., 2013; Wu and Kuo, 2018). Therefore, clinicians and basic scientists have investigated alternate therapeutic methods through the recruitment of modern technologies. In recent decades, tissue engineering has attracted increasing widespread attention, and different tissue engineering strategies for the reconstruction of the bladder have been studied (Figure 2E; Serrano-Aroca et al., 2018).

Numerous scaffolds and biomaterials have been studied as new tissue engineering platforms in terms of safety, feasibility, and efficiency in the regeneration of the bladder wall. Naturally derived collagen-based scaffolds were among the first choices owing to their favorable biocompatibility and natural essence. These scaffolds are the ECM of tissue after the removal of cellular components by mechanical and/or chemical means. Naturally derived scaffolds are very rich in structural proteins of ECM which offer unlimited cell recognition sites for implanted and immigrating cells. In addition, these scaffolds retain a great range of growth factors that can influence cells’ viability, fate determination, and remodeling of new forming tissue (Byron et al., 2013; Brown and Badylak, 2014). Importantly, cells are capable of remodeling and degradation of ECM with a broad array of enzymes and mechanisms (Lu et al., 2011). The ECM based scaffolds are usually prepared from small intestinal submucosa (SIS), bladder matrix, amniotic membrane, pericardium and, dermis of animal or human origins (El-Kassaby et al., 2003; Sartoneva et al., 2010). However, some of their features may hamper their use in tissue engineering applications, especially bladder tissue engineering. Naturally derived scaffolds cannot be tailored conveniently to the desired features for different applications in diverse contexts of tissue engineering (Brovold et al., 2018). Different preparation and sterilization methods, age, and species of the donor animals in the processing and production of natural scaffolds significantly affect the physical and biochemical properties of these scaffolds, resulting in considerable batch-to-batch dissimilarity. This point raises a great level of uncertainty when the outcomes of different animal and clinical studies are tried to be compared (le Roux, 2005; Zhang et al., 2006; Caione et al., 2012; Zhang and Liao, 2014; Liu et al., 2017). Furthermore, naturally derived scaffolds are generally prepared from allogeneic or xenogenic sources; so, the remained cellular components and matrix proteins after the decellularization process may have the potential of immunogenic reactions [for review, please see Wong and Griffiths (2014)]. The mechanical feature of naturally derived scaffolds is another limiting factor when special biophysical prerequisites are needed. Regarding the bladder as a load-bearing organ, the implanted scaffold has to withstand the repetitive filling-emptying cycles (Schaefer et al., 2013). In addition, the high density of collagen fibers in naturally derived scaffolds hinders efficient infiltration of cells and consequently limits their cell loading capability (Liu et al., 2009).

Synthetic scaffolds on the other hand, offer more versatile and tailorable properties to meet the requirements of target tissues. Natural and synthetic polymers can be utilized for fabrication of synthetic scaffolds. During the fabrication and post-processing of synthetic scaffolds, the mechanical features, porosity, biodegradability, and integration of bioactive molecules are controllable. Although favorable biocompatibility, degradability, vast availability and low toxicity of natural polymers are critical features in scaffold construction, their batch-to-batch variation, complex structure and, intricate extraction process may hurdle their application (Muhamad et al., 2014). Concerning synthetic polymers, lack of cell-recognition sites, damaging effects of degradation products, and *in vivo* high durability associated with foreign-body reactions may limit their applications (Wolfe et al., 2011; Wolf et al., 2015). However, their controllable and scalable production as well as versatile usability in different fabrication methods would offer various opportunities in their usage. Natural (e.g., collagen, silk, and chitosan) and synthetic [polycaprolactone (PCL), poly-L-lactic acid (PLLA), poly(lactic-co-glycolic acid) (PLGA), and Poly(lactide-co-caprolactone) (PLCL)] biomaterials have been utilized for the fabrication of synthetic scaffolds in bladder tissue engineering (Lin et al., 2015; Serrano-Aroca et al., 2018). Furthermore, through innovative fabrication methods, composite and hybrid scaffolds can be designed to take the advantage of different biomaterials in the same context (Atala et al., 2006; Eberli et al., 2009; Horst et al., 2013). This idea is very valuable when different cells have to be transplanted, or different layers with distinct features in the same scaffold are demanded.

Biodegradation and bioresorbability rate of constructs in urologic tissue engineering are very critical parameters for achieving favorable outcomes, regardless of the natural or synthetic origin of biomaterials. The degradation products should not exert toxic and inflammatory effects and their concentration (based on degradation rate) should be tolerable for regenerating and adjacent tissue. In addition, these products have to be easily removable through physiological metabolic processes (Bergsma et al., 1995; Azevedo and Reis, 2005). *In vivo* application of non-degradable materials could be associated with foreign-body responses, stone formation, contractures, and non-functional remodeling of regenerated tissue (Falke et al., 2000; Kim et al., 2000; Abbas et al., 2019). On the other hand, biodegradable scaffolds can provide a constructive matrix for neo-tissue formation and regeneration. Degradation of these materials inhibit prolonged exposure of urine to foreign material. Synchronous rate of scaffold degradation with neo-tissue formation is highly critical for a functional successful regeneration; otherwise, accelerated degradation of scaffold can compromise the mechanical integrity of scaffold and may result in urine permeation to deeper layers of tissue that can be associated with reconstitution failure.

The beginning of the third millenium was accompanied by promising and impressive advances for the tissue engineering of the bladder. Two consecutive reports revealed that tissue-engineered constructs successfully restored the bladder tissue and function in cystectomy models of big animals (Yoo et al., 1998; Oberpenning et al., 1999). These findings established the cornerstone of upcoming trials in human subjects (Atala et al., 2006; Caione et al., 2012; Joseph et al., 2014; Zhang and Liao, 2014). Atala and colleagues in their pioneer work performed augmentation cystoplasty by tissue engineering in seven patients with myelomeningocele (Atala et al., 2006). They isolated and propagated human bladder cells (urothelial cells, UCs, and smooth muscle cells, SMCs) from tissue biopsies and applied them autologously for preparation of tissue constructs, but the same procedure was not followed for the fabrication of all the tissue scaffolds. Although modest improvement in bladder capacity and compliance were noticed, no severe complications were reported after 22–61 months of follow-up. This study shaped a motivating landscape for more studies in the field of reconstructive medicine. However, a phase 2 clinical trial by Joseph and colleagues used poly(glycolic acid) (PGA)/Polylactic acid (PLA) scaffold pre-seeded with autologous bladder cells for bladder augmentation in ten patients with spina bifida (Joseph et al., 2014). Unfortunately, patients did not show any significant clinical and statistical improvement in the functional indices of the bladder after 12–36 months of follow-up. More importantly, severe adverse events such as bowel obstruction, bladder rupture, and pelvic obstruction occurred in some patients, which caused major reservations regarding the safety and efficiency of this method. Another human trial by Caione and colleagues utilized a commercial available SIS (Surgisis^®^, Cook Urological, Spencer, IN, United States) for augmentation of the bladder in five patients with exstrophy–epispadias complex (Caione et al., 2012). Although the transplanted SIS marginally supported bladder regeneration and slightly enhanced the bladder capacity and compliance, it failed to present significant clinical benefit. With the same strategy, Zhang and coworker, augmented the bladder in eight patients and surprisingly revealed that after a mean follow-up of 12 months (range from 11–36 months) the reconstructed bladders possessed significantly higher capacity and compliance compared to preoperative amounts (170.1 ± 75.7 ml vs. 385.5 ± 52.8 ml, and 5.9 ± 4.0 ml/cm H_2_O vs. 36.3 ± 30.0 ml/cm H_2_O, respectively) (Zhang and Liao, 2014). SIS provides a biocompatible matrix with no adverse reaction in cross-species transplantations, and it is a supportive matrix for cell growth and regeneration; however, the intrinsic drawbacks associated with natural scaffolds have hindered its clinical applications [for review, please see Lin et al. (2013)]. Although the Food and Drug Administration has approved SIS for some clinical reconstructive applications, its efficiency and suitability for bladder augmentation is questionable because of inconsistent findings from animal studies and human trials.

## Electrospinning for Bladder Tissue Engineering

Various electrospun scaffolds have been constructed with different biomaterials, fabrication methods, and designs for bladder tissue engineering so far. The fibrous structure of electrospun scaffolds provides an ECM-resembling matrix for cells, resulting in an ideal substrate for cells’ growth and differentiation. Besides, the electrospinning procedure can be tailored in a way to achieve fibers with nano-sized dimensions which is proved to have several important advantages for bladder tissue engineering including increased cell attachment, proliferation, regeneration, ECM components production, and inhibition of calcium oxalate stone formation (Thapa et al., 2003a,b; Pattison et al., 2005; Chun et al., 2009; Yao et al., 2013). Generally, electrospun scaffolds in the context of bladder tissue engineering can be categorized based on the complexity of their composition into simple, composite, and hybrid scaffolds (Figures 1B–G).

Simple electrospun scaffolds contain a single biomaterial in the structure of the fibers (Figure 1B). In *in vitro* studies, electrospun scaffolds can be an appropriate substrate for investigating cells’ behavior *ex vivo* and provide a useful tool for basic studies regarding differentiation status and the effects of bioactive agents on the cells. In this regard, it has been shown that electrospun scaffolds of PLLA and PLGA were capable of supporting the attachment and proliferation of human bladder cells (Sharifiaghdas et al., 2014; Derakhshan et al., 2016). Besides, the addition of collagen to the PLGA scaffold after the electrospinning significantly improved its cell attachment feature when compared with the natural AM membrane (Sharifiaghdas et al., 2014). Uchida et al. showed that the layer-by-layer addition of fibronectin and gelatin (FN-G) on electrospun poly (carbonate-urethane) urea (PCUU) could increase cell adhesion and migration of bladder SMCs. The PCUU^FN–G^ scaffold also supported UROtsa cells, human urothelium-derived cell line, for the formation of a multilayer structure (Uchida et al., 2016). In one study by Baker et al., polystyrene, which is routinely used for the manufacturing of tissue culture vessels, was employed for electrospinning which demonstrated acceptable mechanical strength and biocompatibility. In addition, the alignment of fibers in the electrospun polystyrene was conducive for cultured human bladder SMCs, as oriented them parallel to the fibers axis in the aligned scaffold (Baker et al., 2006). Fibrinogen, an integral component of the blood clot, was also recruited for the preparation of scaffold through electrospinning. It was demonstrated that nanofibrous fibrinogen provided a supportive condition for human bladder SMCs proliferation and migration, and could be remodeled by seeded cells (McManus et al., 2007). Another study showed that bacterial derived poly [(R)-3-hydroxybutyrate] (PHB) could be processed as an electrospun scaffold. Plasma treatment of PHB electrospun scaffold resulted in decreased calcium oxalate deposition and enhanced biocompatibility for uroepithelial cells (Karahaliloğlu et al., 2016). Culture of cells/stem cells on the surface of electrospun scaffolds sends more physiologic signals from the substratum to the cells than culture vessels surface; these signals can affect gene expression regulation and profile of transcripts (Yin et al., 2015; Kennedy et al., 2017). The culture of induced pluripotent stem cells on the electrospun scaffolds of PLGA increased the expression of smooth muscle-specific markers when compared with cells cultured on the tissue culture polystyrene (Mirzaei et al., 2019). Similarly, it has been revealed that human adipose tissue-derived mesenchymal stem cells cultured on polyvinylidene difluoride (PVDF) electrospun scaffold, upregulated the expression of smooth muscle-specific markers (Ardeshirylajimi et al., 2018).

Although such studies may build a primary notion for designing more complex experiments, the functionality of scaffolds without animal study cannot be appraised properly. Moreover, owing to the complex function and regeneration process of the bladder, animal studies in this area have to be designed with important considerations. Sivaraman et al. compared the mechanics of three elastomer-based electrospun scaffolds [polyglycerol sebacate–polycaprolactone (PGS-PCL), poly (ether-urethane) urea (PEUU) and, PCUU] and found the PCUU scaffold as the most resembling scaffold with bladder mechanics. Then, they employed the PCUU scaffold for bladder augmentation in a bladder outlet obstruction rat model. After 3 weeks of follow-up, increased survival, bladder capacity, and voiding volume were observed (Sivaraman et al., 2019). Pokrywczynska et al. evaluated the applicability of PLCL scaffold in the rat model. PLCL scaffold was seeded with rat adipose-derived stem cells and implanted in the bladder for 12 weeks. They reported negative results as the reconstruction failed after perforation of scaffold and formation of fistula (Pokrywczynska et al., 2014). These findings may be connected to the mechanical features of the scaffold and/or the asynchrony between the degradation rate of PLCL and new tissue remodeling. However, Shrestha et al. (2019) fabricated a similar scaffold with some modifications that produced considerably different results. The PLCL scaffold was cultured with human adipose-derived stem cells, and a multilayer scaffold was prepared by stacking five sheets of the pre-seeded scaffold. They reported that rat bladder reconstruction with a multilayered scaffold after 4 weeks showed increased function and higher contractility of the regenerated smooth muscle layer. This study underlines that the mechanical features of scaffolds are as critical as biocompatibility and cell supporting properties for bladder tissue regeneration. In this regard, the electrospun silk fibroin scaffold was mechanically reinforced by stretching in ethanol and used for bladder tissue engineering in rabbit. Although the investigators did not follow a cell-based strategy, after 8 weeks, cell layers expressing related markers were restored. Nevertheless, caliculi formation and inflammatory reactions were also reported (Huang et al., 2016; Table 1).

**TABLE 1:** Pre-clinical studies utilizing electrospun scaffolds for tissue engineering of bladder.

**Study**	**Biomaterial**	**Electrospinning method**	**Fiber Diameter (nm)**	**Solvent**	**Cell Source**	**Animal Model**	**Scaffold Shape (and Size)**	**Follow-up time**	**Outcome**
Feng et al. (2019)	PLCL PLCL/HA	Coaxial-electrospinning	784.2 ± 138.2	HFIP/formic acid	None	Rat	Dome-shaped (40–50% supratrigonal cystectomy)	10 weeks	Smooth muscle regeneration Increased bladder capacity Stone formation
Shrestha et al. (2019)	PLCL	Single-jet	NA	HFIP	hADSCs	Rat (detrusor smooth muscle-removed bladder model)	Sheet (1*1 cm)	4 weeks	Significantly increased compliance and higher smooth muscle contractility of regenerated tissue in the pre-seeded group
Sivaraman et al. (2019)	PCUU	Single-jet	NA	HFIP	None	Rat (bladder outlet obstruction)	Sheet (1*1 cm)	3 weeks	Increased voiding volume and caliculi formation
Horst et al. (2017)	Hybrid PEU/BAM Hybrid PLGA/BAM	Double-jet electrospinning of either PEU or PLGA with PEG (porogen) directly onto BAM	PEU: 3600 ± 300 PLGA: 4700 ± 300	HFIP/chloroform for PEU Chloroform for PLGA	B-SMCs	Rat	Sheet (50% cystectomy)	8 weeks	Pre-seeded hybrid PLGA/BAM showed marginally superior functional bladder indices Greater regenerative histological score in the pre-seeded hybrid PEU/BAM
Shakhssalim et al. (2017)	PCL/PLLA	Double-jet	120–1500	Chloroform/DMF	B-SMCs B-UCs	Dog	Circular (10 cm^2^)	12 weeks	Suitable cyto-architecture and expression pattern of markers in regenerated tissue in the smooth muscle pre-seeded scaffold No caliculi formation
Huang et al. (2016)	Silk fibroin	Single-jet	NA	Water	None	Rabbit (detrusor smooth muscle-removed bladder model)	Sheet (1.5*2 cm)	8 weeks	Restoration of tissue layers in the regenerated region with the expression of pertinent markers Caliculi formation Mild acute and chronic inflammatory reactions
Pokrywczynska et al. (2014)	PLCL	Single-jet	1390 ± 320	Chloroform/DMF	rADSCs	Rat	Sheet (1 cm^2^)	12 weeks	A general failure of PLCL scaffold due to perforation, and formation of fistula and diverticula
Horst et al. (2013)	Hybrid PLGA/BAM	Single-jet electrospinning of PLGA directly onto BAM	4500 ± 250	Chloroform	None	Rat	Sheet (50% cystectomy)	8 weeks	Regeneration of bladder wall with proper cyto-architecture Hybrid scaffold maintained normal bladder capacity

*PLCL, poly(L-lactide-co-caprolactone); PLCL/HA, poly(L-lactide-co-caprolactone)/hyaluronic acid; HFIP, 1,1,1,3,3,3-hexafluoro-2-propanol; NA, not assessed; hADSCs, human adipose-derived stem cells; PCUU, poly (carbonate-urethane) urea; PEU/BAM, polyesterurethane/bladder acellular matrix; PLGA/BAM, poly(lactic-co-glycolic acid)/bladder acellular matrix; PEU, polyesterurethane; PLGA, poly(lactic-co-glycolic acid); PEG, poly(ethylene glycol); BAM, bladder acellular matrix; B-SMCs, bladder smooth muscle cells; PCL/PLLA, poly(ε-caprolactone)/poly(L-lactic acid); DMF, N,N-dimethylformamide; B-UCs, bladder urothelial cells; rADSCs, Rat adipose derived stem cells. NA.*

The possibility of modifying and adjusting different production parameters during the electrospinning process offers a great opportunity for creating scaffolds with diverse features for quite different applications. Concurrent application of two or more biomaterials in a single scaffold is a simple way for the fabrication of new scaffolds with different characteristics from those composed of either of the biomaterials. This strategy can be performed by the simultaneous spinning of different biomaterials through multiple jet electrospinning, mixing biomaterials in a solvent in single jet electrospinning, and coaxial electrospinning (Figures 1C–E). Construction of composite scaffolds presents a more pronounced diversity in the structure and application of electrospun scaffolds through incorporating different features of biomaterials in the same matrix. It has been revealed by Shoae-Hassani et al. (2015) that cultivation of human endometrial stem cells on the composite electrospun scaffolds of silk/collagen when compared to the silk scaffold could improve the expression of urothelial specific markers (uroplakin-Ia, uroplakin-Ib, uroplakin-II, uroplakin-III, and cytokeratin 20) during their differentiation process into UCs. Considering the fact that ECM matrix comprises important structural and signaling molecules for cells in their native environment. It would be a perfect means if ECM be reconfigured into a scaffold for tissue engineering purposes as it contains critical cues for target cells that can control and improve the regeneration process. Li et al. prepared powder of porcine acellular bladder matrix composed of key ECM proteins such as collagen IV, laminin, and fibronectin. They showed the possibility of the construction of a composite electrospun scaffold of ECM with micro-nano fibers. This scaffold presented favorable mechanical characteristics and cyto-biocompatibility which suggest its potential applicability in *in vitro* assays and tissue engineering (Li et al., 2018). In this regard, Stankus et al. fabricated composite electrospun scaffolds by mixing powder of porcine acellular bladder matrix and synthetic biodegradable elastomer, PEUU, in a single solution at different proportions. It was observed that increasing the amounts of PEUU in the composite scaffold was associated with higher tensile strength and breaking strain, but the higher percentage of ECM enhanced the SMCs’ behavior through increasing the cells adhesion and proliferation (Stankus et al., 2008). The composite electrospun scaffold of PCL/PLLA was utilized in a series of studies for bladder tissue engineering. Firstly, it was demonstrated that PCL/PLLA scaffold could support the viability and proliferation of human and dog bladder cells. Cells on this scaffold maintained the expression pattern of their specific markers (Shakhssalim et al., 2013; Naji et al., 2015). This scaffold also provided a convenient platform for evaluating the effect of mechanical stimulations on bladder cells (Ahvaz et al., 2012, 2013), which has been previously proposed to play critical roles in realizing the normal behavior of bladder cells and should be considered for making more functional tissue constructs for bladder tissue engineering (Backhaus et al., 2002; Farhat and Yeger, 2008; Bouhout et al., 2011).

**FIGURE 3:**
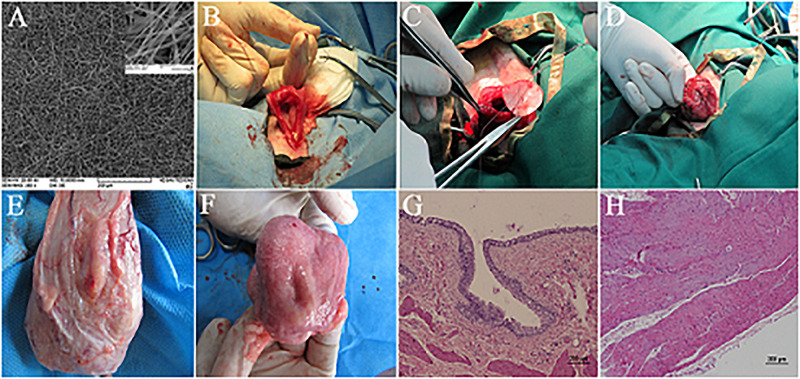
Bladder reconstruction using electrospun PCL/PLLA scaffold in canine model. **(A)** SEM micrograph of PCL/PLLA scaffold with fiber diameter in the range of 120 to 1500 nm. **(B–D)** Surgical technique of PCL/PLLA scaffold implantation. **(E)** External and **(F)** luminal view of reconstructed bladder in smooth muscle transplanted group after 3 months, with full coverage of implanted scaffold. Hematoxylin–eosin (H&E) staining of regenerated area in smooth muscle transplanted group representing the formation of well-developed urothelium **(G)** and smooth muscle layer **(H)**. Reprinted with permission from Elsevier Ltd., Shakhssalim et al. (2017).

Animal studies by Shakhssalim et al. utilized the PCL/PLLA scaffold for the reconstruction of the bladder in dogs. They reported that the scaffolds pre-seeded with autologous bladder SMCs, after 3 months of regeneration, entirely integrated with native tissue and ingrowth of new forming tissue from the edges of construct restored the cell layers (Shakhssalim et al., 2012, 2017; Figure 3). They also conducted a cell tracking experiment by using a lentiviral expression of reporter genes in transplanted cells [green fluorescent protein (GFP) and jellyfish red fluorescent protein (JRed) for urothelial and smooth cells, respectively]. However, the investigators did not detect any signal of reporter genes in regenerated tissues, though the effect of scaffold pre-seeding, especially for SMCs, was evident. It was concluded that the transplanted cells might create a transient feeder layer, which accelerates and support the formation of new tissue from native bladder tissue, as previously observed and proposed (Turner et al., 2011). Synthetic polyesters are among the popular biomaterials for electrospinning, but lack of cell recognition signals in these biomaterials demands innovative modifications during the fabrication process to insert cell recognizable groups. Feng et al. utilized the coaxial-electrospinning method to coat PLCL electrospun nanofibers with hyaluronic acid (HA) (an ECM component). They reported that composite PLCL/HA scaffold was more hydrophilic with increased anisotropic wettability and swelling. The HA coating also increased the proliferation and migration of SMCs on PLCL/HA scaffold. Bladder reconstruction with dome-shaped PLCL/HA scaffold in rat revealed promising smooth muscle layer regeneration and increased capacity of the tissue-engineered bladder (Feng et al., 2019). Del Gaudio et al. used a combination of PCL and poly(3-hydroxybutyrate-co-3-hydroxyvalerate) (PHBV) (50:50) for the fabrication of an electrospun scaffold for bladder tissue engineering in rat. Although they showed that the cell layers were restored after 12 weeks of regeneration, the small size of constructs in these kinds of studies restricts the precise evaluation of scaffolds for bladder reconstruction (Del Gaudio et al., 2013; Table 1).

Different fabrication and post-production techniques can result in scaffolds with significantly distinct characteristics and applications, even from a single biomaterial. In this regard, a scaffold can be constructed via a combination of methods, which ends in a hybrid scaffold with a layered structure. Each layer of a hybrid scaffold keeps distinct features, which provides unlike scaffolding faces for different cells of a complex tissue (Figure 1F). In addition, some functions, such as being an effective barrier, can be easily developed in the structure by dedicating a layer to the specific function without influencing the major role of other layers. Kundu et al. employed a spin-coating method for the construction of thin films from PCL and PLLA and then utilized them as a substrate for direct electrospinning of PCL onto the films. They showed that the resulted hybrid scaffolds supported the attachment and progressive proliferation of UCs while created an impermeable barrier against small molecules thanks to the film layer in the hybrid construct (Kundu et al., 2011). Another study introduced an electrospun scaffold of PCL to reinforce the weak mechanical features of freeze-dried bladder ECM sponge. To this end, PCL scaffold was embedded in rat acellular bladder ECM hydrogel and then the construct was freeze-dried. The resultant hybrid scaffold indicated considerably improved mechanical strength compared to ECM sponge, and contrary to the sponge it could be utilized in surgery (Ghafari et al., 2017). Using a relatively similar strategy, Ajalloueian et al. constructed a hybrid scaffold with electrospun PLGA and collagen. The PLGA scaffold was placed between two layers of collagen gel and the construct was unified by compression. The hybrid construct showed significantly increased tensile strength in comparison to the collagen gel (3.57 MPa vs. 0.6 MPa). Through this compression method, it was possible to integrate minced bladder mucosa into the hybrid scaffold. It was demonstrated that cells from minced tissues could migrate, proliferate, and re-organize into multilayered urothelium after 4 weeks *in vitro* (Ajalloueian et al., 2014).

Horst et al. fabricated a hybrid scaffold by direct electrospinning of PLGA onto the abluminal surface of the porcine bladder acellular matrix (BAM). The structural integrity of the hybrid scaffold was stabilized by continuous rehydration of BAM during the electrospinning procedure. The functionality of the hybrid scaffold for bladder reconstruction was compared with BAM in a rat model of bladder partial cystectomy (50%). After 8 weeks, the restoration of bladder urothelium and smooth muscle layers was reported. Moreover, the bladder capacity was maintained at the normal level in the hybrid scaffold implanted group compared to the distended bladders of the BAM group (Horst et al., 2013). In a following study, the hybrid scaffold of PLGA/BAM was modified by increasing the porosity of the electrospun layer. Polyethylene glycol (PEG), as a porogen, was integrated into the electrospun layer during the fabrication process. Water-soluble PEG was then washed out after the spinning procedure by overnight incubation in phosphate buffered saline, leaving more and bigger pores on the fibrous structure. Evaluation of the modified hybrid scaffold in the rat bladder cystectomy model showed a higher density of microvessels formation after 4 weeks (Horst et al., 2014). Another study by the same group compared the hybrid scaffold of PLGA/BAM with poly(ester urethane) (PEU)/BAM. PEG was utilized for increasing the porosity in the fibrous part of both scaffolds. In addition, the scaffolds were seeded with bladder SMCs before implantation and compared with bare scaffolds. Following 8 weeks of regeneration in a rat bladder cystectomy model, they reported better tissue healing and regeneration in pre-seeded PEU/BAM group with less inflammatory responses (Horst et al., 2017; Table 1).

Considering three classes of solvents based on ICH (International Conference on Harmonization of Technical Requirements for Registration of Pharmaceuticals For Human Use) guideline (Guideline, 2005), most of studies in the context urologic tissue engineering by electrospun scaffolds have utilized class 2 solvents (solvents to be limited). In addition, the quantification of residual solvent in the electrospun mat has been rarely studied (Nam et al., 2008). The ultimate focus of researchers on the structural features of nanofibers and challenging process of optimization with novel solvents may reason this critical point to be neglected. The type of solvent in the electrospinning process would exert concerns regarding final application of electrospun scaffolds (pharmaceutical or reconstructive applications) and environmental and personnel exposure. Consequently, the concept of “Green Electrospinning” has been introduced since 2010 (Sun et al., 2010; Krishnan et al., 2013) which implicates the use of class 3 solvents (benign solvents, low toxic potential) for electrospinning. Green electrospinning has relived concerns about the toxic effects of electrospinning solvents for their final application and environmental issues. Hence, the number of works utilizing this strategy has increased recently (Liverani et al., 2017) and, future works dedicated to clinical translation of electrospun scaffolds should consider this concept. Application of electrospun products as medical devices without inclusion of human or animal cells are supported with well-established regulatory framework, however; cell-based reconstructive strategies utilizing electrospun scaffolds require more complicated certificates (Wilson, 2011; Burke et al., 2017). Although clinical transition of electrospun products in the field of reconstructive urology is still awaiting for more in-depth studies, promising vistas can be visioned for the application of electrospun scaffolds in reconstruction of urologic tissues.

Besides, scale-up and robustness of electrospinning are critical challenges specially when converting an electrospun construct from R&D to a manufacturing scale (Burke et al., 2017; Liu et al., 2020). Low productivity of electrospinning process (0.01–1 g/h) does not meet the requirements of high throughput production of commercial and clinical scales. Consequently, upscaling of electrospinning has been investigated through nozzle-type modifications and free surface technologies [for review, please see Vass et al. (2019)].

## Urethral Tissue Engineering

The urethra is a narrow fibromuscular tubular tissue connecting the urinary bladder to the urinary meatus that is responsible for urine removal. In males, the urethra is much longer than females (20 vs. 4 cm) and also carries semen. The male urethra is divided into different segments: prostatic urethra and membranous urethra (posterior urethra), and bulbar urethra, penile urethra, and fossa navicularis (anterior urethra) (Eylert and Nambiar, 2019). The male urethra, after leaving the bladder, passes through the prostate gland and then goes into the penis and travels its entire course to meatus embedding in the center of penile corpus spongiosum. The female urethra is a shorter tubular structure that lies directly behind the symphysis pubis, embedded in the anterior wall of the vagina (Jung et al., 2012). In both sexes, the epithelial lining of urethra changes from proximal transitional epithelium to pseudostratified columnar in the middle and finally into stratified squamous in distal regions. In the membranous urethra of males, the muscle layer comprises a smooth muscle sphincter and a striated muscle sphincter, which controls the passage of urine or semen. A circular layer of striated muscle surrounds the smooth muscle layer of the female urethral wall around the urethra at the level of the pelvic floor and creates the sphincter with horseshoe shape (Figure 2C; Jung et al., 2012).

Stricture of the urethral wall is a common and challenging urological condition, affecting 0.2 to 0.9% of men (Lumen et al., 2011; Rourke and Hickle, 2012). The high prevalence and recurring rate of urethral stricture produce a significant economic and disease burden. Urethral strictures usually can result from trauma (such as straddle injury), tumor resection, infection, congenital malformation, iatrogenic injuries, and hypospadias surgery (Lumen et al., 2009; de Kemp et al., 2015; Verla et al., 2019). Stricture formation is the consequence of dramatic changes in tissue composition following fibrosis and extensive collagen deposition, which end in narrowing of the urine passage route. It usually manifests with urinary infection, incontinence, and overactive bladder symptoms (Nuss et al., 2012; Verla et al., 2019). The surgical treatment method of urethral stricture relies on length, location, severity, and recurrence history of the stricture (Verla et al., 2019). Local and confined strictures are generally managed by dilation of the urethra at the affected site. Urethrotomy, excision of stricture and direct rejoining by end-to-end anastomosis, is applied for more severe injuries. However, the complex and long defects often require the incorporation of a graft or a flap in the site of injury to augment the stenotic segment. Clinically, the currently available grafts for complex long-segment urethral defects are autologous grafts, including penile flap, bladder mucosa, oral mucosa, colonic mucosa, and tongue mucosa (Peterson and Webster, 2004). Nevertheless, the use of autologous grafts is associated with several complications such as limited donor sites and tissues, the need for additional harvest, and the probability for donor site morbidity (Pederzoli et al., 2019). Tissue engineering as an alternative approach, holds the potential of addressing these limitations using autologous cells and biodegradable scaffolds (Figure 2F; Simsek et al., 2018a; Rashidbenam et al., 2019). An ideal scaffold for urethral tissue engineering should be biocompatible, biodegradable, and own suitable three dimentional microstructure and biomechanical attributes to promote the proliferation of seeded cells and ingrowth of native tissues when was implanted. The scaffold should have enough strength to tolerate the mechanical forces exerted during the surgery and at the same time, should have a matched mechanical compliance to the native urethra to stretch and recoil during penile erection and dilate the lumen during micturition (Selim et al., 2011; de Kemp et al., 2015; Zhang et al., 2015; Simsek et al., 2018b; Rashidbenam et al., 2019). In addition, the scaffold should also act as an impermeable barrier to preserve the underlying tissues from the toxic components of urine (de Kemp et al., 2015).

Numerous synthetic and natural matrices, with diverse fabrication methods and post-production treatments, have been utilized for urethral tissue engineering to date. In this area, tissue-engineered constructs with tubular (seamless), tubularized, and onlay configurations in conjunction with stem cells or progenitor cells have been studied in animal studies and human trials (Pederzoli et al., 2019). Although several works have reported promising outcomes in animal and human studies, the design and construction of an engineered biomimetic construct which meets all requirements for the regeneration of long and complex urethral strictures is still a challenge for researchers of regenerative medicine.

Similar to many organs treated by tissue engineering methods, collagen-based naturally derived scaffolds were among the first preferred options for reconstruction of urethra. This is because of the superior biocompatibility of these materials, and their supportive nature for cell attachment, growth, colonization, and remodeling of regenerating tissues (El-Tabey et al., 2012; Orabi et al., 2013; Sayeg et al., 2013; Wang et al., 2014; Liu et al., 2017). El-Tabey et al. used a canine acellular bladder matrix, pre-seeded with autologous bladder cells (UCs and SMCs), for urethroplasty of 3 cm segment of the urethra in female dogs, and followed the regeneration process for 11 months. Although the small number of animals in each time point of analysis was a major limitation of this study, narrowing of urethra and stricture were noticed in most of the animals, which could be related to marked shrinkage, fibrosis, and deficient urothelial layer establishment in regenerated tissue (El-Tabey et al., 2012). Another study by Orabi et al. applied tubularized porcine acellular bladder matrix for the regeneration of a clinically relevant (6 cm) segment of the urethra in male dogs. Autologous bladder cells were isolated and cultured on the acellular matrix, and its regenerative potential was compared with the bare matrix in a follow-up period of 12 months. The pre-seeded matrix supported the reconstruction of a wide-caliber urethra without stricture, while the bare matrix failed (Orabi et al., 2013). Contrary to the previous study, the onlay application of bladder submucosa pre-seeded with autologous SMCs in rabbit did not demonstrate any significant regenerative advantage over the bare matrix, and both of them resulted in fully restored normal architecture of the urethra (Sayeg et al., 2013). In this regard, it has been shown that urine-derived stem cells cultured on SIS held superior capacity for regeneration of urethra. The pre-seeded SIS promoted and supported the progress of regeneration, greater density of vasculature, and higher diameter of the urethra when compared with SIS (Liu et al., 2017). In sum, it seems that the cell-based approach with scaffolding is the most promising strategy for urethral reconstruction through tissue engineering. Since the proper scaffolds for tissue engineering of urethra do not require complex mechanical features as bladder demands, the recruitment of natural scaffolds with weak mechanical strength but strong capability in the promotion of cellular regeneration is more feasible for the urethra. However, the intrinsic drawbacks associated with natural scaffolds motivated the researchers to assess artificial scaffolds’ applicability in this field (FitzGerald and Kumar, 2014; Wong and Griffiths, 2014).

Chung et al. prepared a bi-layer fibroin silk scaffold composed of a porous layer and a compact film layer. The outcome of onlay urethroplasty with this scaffold in rabbit was compared with SIS for 3 months, and it was reported that both scaffolds were equally efficient in urethral reconstruction as no stricture, fistula, and stone were observed in regenerated normal caliber urethras (Chung et al., 2014). Furthermore, in another study by Algarrahi et al., the same scaffold (bi-layer fibroin silk) was used; however, they induced urethral scar formation by electrocoagulation in the animal model to simulate natural histopathological circumstance of patients with urethral strictures. According to retrograde urethrography findings, the implantation of the scaffold was associated with a 39% reduction in stenosis of the induced group, but the smooth muscle layer did not develop completely (Algarrahi et al., 2018).

Collagen-based scaffolds were always among the most interesting options for investigators in different contexts of tissue engineering. There has been a continuous effort for upgrading and optimizing the characteristics of these scaffolds. In this regard, Pinnagoda et al. created a tubular scaffold of rat-tail collagen type I and reconstructed a 2 cm segment of rabbit urethra without cell seeding. After 9 months of follow-up, in 40% of animals fistula or stenosis were observed. The cyto-architecture was gradually developed further by increasing the postoperative regeneration time. The implanted bare scaffold was spontaneously re-populated by urothelium and SMCs with different speeds, 1 and 6 months, respectively (Pinnagoda et al., 2016).

El Kassaby et al., in a comparative study, evaluated the efficiency of human cadaveric acellular bladder matrix for stricture repair in human and compared the outcomes with standard urethroplasty with buccal mucosa graft. They found that the quality of urethral bed (i.e., absence of spongiofibrosis, which is directly related to the history of previous interventions) has a significant role in the performance of implanted bare scaffold. They reported that eight of nine patients with an intact, healthy urethral bed showed patent grafts, while two of six in unhealthy cases revealed patency (el-Kassaby et al., 2008). These findings emphasize that the findings of animal studies, which mainly achieved from normal animals, cannot be easily translated into clinical applications. El Kassaby et al., in another study, which is the largest human trial for urethral tissue engineering, used the human cadaveric acellular bladder matrix for reconstruction of anterior urethral strictures (1.65–16 cm) in human subjects aged between 22 to 61 years old. Successful outcomes in 24/28 patients were reported according to retrograde urethrography after a mean follow-up of 37 months; the other 4 patients that developed re-stricture at the anastomotic site were capable of voiding after endoscopic incision (El-Kassaby et al., 2003). In addition, one study by Le roux reported an ineffective application of SIS for endoscopic urethroplasty of bulbar urethral strictures in nine patients (le Roux, 2005).

The cell seeding approach was also examined in human trials. PGA:PLGA scaffold, which was previously reported to be suitable for bladder reconstruction (Oberpenning et al., 1999), was employed by Raya-Rivera et al. for urethroplasty of posterior urethral strictures in boys after pre-seeding with autologous bladder UCs and SMCs. 4 out 5 patients revealed favorite outcomes in up to 6 years of follow-up (36–76 months). The last patient was managed with an incision without further interventions (Raya-Rivera et al., 2011). Another pediatric study by Fossum et al. (2012) reported promising outcomes, after 6–8 years of follow-up, by using pre-seeded (UCs) acellular dermis for the reconstruction of the urethra in six boys with severe scrotal and perineal hypospadias.

In conclusion, the results of urethral tissue engineering in humans are much more promising than corresponding works in the bladder. This point may be attributed to the less complex structure and function of the urethra in comparison to the bladder. However, these findings can only provide a basis for further future studies concentrating on more elaborate constructs for the regeneration of long and complex urethral strictures via tissue engineering methods.

## Electrospinning for Urethral Tissue Engineering

Electrospinning, thanks to the numerous influential controllable process parameters, can potentially be an ideal method to make a suitable urethral scaffold with desired permeability, porosity, biocompatibility, topography, and mechanical properties (Xie et al., 2013; Wei et al., 2015; Zhang et al., 2015). Several electrospun scaffolds have been studied so far for urethral tissue engineering applications, all of which resulted in promising outcomes (Fu et al., 2009; Selim et al., 2011; Xie et al., 2013; Wei et al., 2015; Zhang et al., 2015; Lv et al., 2016; Simsek et al., 2018b). However, since there is no standard protocol for urethral graft evaluation, these studies generally lack a comprehensive evaluation. Selim et al. evaluated the effect of different sterilization and cell seeding methods on the mechanical properties and contraction of a PLGA electrospun scaffold (Selim et al., 2011). Their study indicated that all three sterilization methods – peracetic acid (PAA), γ-irradiation, and ethanol – reduced the strength and elasticity of the scaffolds. In addition, PAA and ethanol drastically reduced the fiber diameter to less than 50% of their initial value and also the latter proved to be an unreliable sterilization method. Cell culture on the sterilized scaffolds also changed their physical and mechanical properties. Cell culture strengthened the ethanol-sterilized samples while reduced the tensile strength and Young modulus of PAA and γ–sterilized ones. They also found that a sequential cell seeding results in reduced tensile strength and young modulus as compared to simultaneous fibroblast and keratinocyte seeding. After cell seeding, all the samples exhibited the same order of contraction, which may be related to the inherent relaxation of the polymeric scaffolds in an aqueous environment; however, fibroblasts resulted in the maximum contraction of the scaffolds, and the addition of keratinocytes made it less prone to the contraction. In another study to reduce the cell-induced scaffolds contraction, Simsek et al. examined the effect of inhibition of lysyl oxidase, an enzyme which promotes the contraction by crosslinking collagen fibers, on the contraction of cell-seeded scaffolds. Cells on the scaffolds were treated with lysyl oxidase inhibitor, beta-amino-propionitrile. Three different electrospun scaffolds (microfibrous PLA, nanofibrous PHBV, and a micro-/nanofibrous trilayer of PLA-PHBV-PLA), and Euroskin were compared (Simsek et al., 2018b). They cultured oral fibroblasts and oral keratinocytes on the two sides of the scaffolds and observed significant contraction in the PLA and Euroskin grafts. The presence of beta-amino-propionitrile had no adverse effect on the viability of the cells during 28 days of culture; however, it could only reduce the contraction in Euroskin. Finally, they concluded that the trilayer PLA-PHBV-PLA scaffold was the best urethral graft as it favorably supported cell attachment and proliferation, presented the closest mechanical properties to the native tissues, and exhibited least contraction even without the use of drug.

Poly-L-lactic acid is the most used polymeric biomaterial for the fabrication of electrospinning scaffolds for urethral tissue regeneration. In this regard, Wang et al. prepared an electrospun PLLA scaffold that used for reconstruction of a 1 * 0.5 cm segment of rabbit urethra. The scaffold was pre-seeded with rabbit adipose-derived stem cells and compared with the bare scaffold. At 6 weeks after surgery, host tissue completely covered the scaffold and urethrostenosis was observed in both groups, although the frequency of urethrostenosis in the pre-seeded group was significantly lower (Wang D.J. et al., 2015). However, in a more clinically relevant setting, a tubular electrospun scaffold of PLLA was examined for the regeneration of induced post-traumatic urethral stricture in a rabbit model. Autologous urethral UCs were isolated and seeded on the scaffold before implantation. After a relatively long follow-up of 24 weeks, the reconstruction outcomes were promising as no stricture and voiding difficulty were observed, and there were no differences in bladder capacity and urethral pressure with the control group (uninjured animal) (Fu et al., 2009). Silk fibroin is another popular natural biomaterial that is owing to its biocompatibility, low price, and versatility for being processed for various fabrication methods, has been evaluated for tissue engineering of different organs (Holland et al., 2019). Silk fibroin based scaffolds with different fabrication and processing techniques have been utilized for the reconstruction of urologic tissues (Sack et al., 2016). Electrospun silk fibroin scaffold was prepared by Xie et al. for the reconstruction of the urethra in dog. After production, the silk scaffolds were stretched in 90% ethanol to intensify their mechanical strength for *in vivo* study. Autologous dog bladder UCs were isolated, and the stretched electrospun silk fibroin scaffolds were pre-seeded before implantation. The dorsal urethra was reconstructed with this scaffold and assessed after 24 weeks. It was demonstrated that animals in the reconstructed groups possess wide caliber urethra without stricture and fistula. Also, histological analysis revealed the formation of a well-developed urothelium layer (Xie et al., 2013; Table 2).

**TABLE 2:** Pre-clinical studies utilizing electrospun scaffolds for tissue engineering of urethra and ureter.

**Urethra**									
**Study**	**Biomaterial**	**Electrospinning Method**	**Fiber Diameter (nm)**	**Solvent**	**Cell Source**	**Animal Model**	**Scaffold Shape (and Size)**	**Follow-up time**	**Outcome**
Lv et al. (2016)	PLLA/PEG	Single-jet	500–1500	Chloroform/DMF	hAMSCs	Rabbit	Onlay (2*1.5 cm)	12 weeks	No stricture and fistula Formation of a multilayered urothelium, and alignment of smooth muscle cells along the scaffold fibers in the pre-seeded group
Wang D.J. et al., 2015	PLA	Single-jet	NA	Dichloromethane	ADSCs	Rabbit	Onlay (1*0.5 cm)	6 weeks	The incidence of urethrostenosis in the pre-seeded group was 50% lower than bare scaffold Formation of multilayered urothelium with organized, smooth muscle cells with lower inflammation in the pre-seeded group
Zhang et al. (2015)	PLCL/Collagen loaded with ICG-001 (Wnt pathway inhibitor)	Coaxial	457 ± 82	2,2,2-Trifluoroethanol/DMSO	B-UCs	Rabbit	Tubularized (2*1 cm)	12 weeks	Wide caliber urethra without strictures and fistulas Less ECM deposition, and more developed smooth muscle and epithelium layers
Xie et al. (2013)	Silk fibroin	Single-jet	800–1200	Water	B-UCs	Dog	Onlay (3.2*1.2 cm)	24 weeks	Wide caliber urethra without signs of stricture and fistula Well-developed stratified urothelial cells in the tissue-engineered group
Fu et al. (2009)	PLLA	Single-jet	3000–5000	Chloroform	Rabbit urethra urothelial cells	Rabbit	Tubular (variable based on the size of stricture induced by PTUS)	24 weeks	Complete coverage of urothelial cells No stricture, voiding difficulty, and other complications

**Ureter**									
Kloskowski et al. (2014)	PLCL	Single-jet	810–2150	Chloroform/DMF	None	Rat	Tubular (1 cm)	4 weeks	Regeneration of urothelium Focal regeneration of smooth muscle layer Hydronephrosis and ureter extension Patency of anastomosis site in 4 out of 6 animals

*PLLA/PEG, poly(L-lactic acid)/poly(ethylene glycol); DMF, N,N-dimethylformamide; hAMSCs, human amniotic mesenchymal cells; PLA, Poly(Lactic Acid); ADSCs, adipose tissue-derived stem cells; DMSO, dimethyl sulfoxide, B-UCs, bladder urothelial cells; PLLA, poly(L-lactic acid); PTUS, post-traumatic urethral stricture; PLCL, poly(L-lactide-co-caprolactone).*

**FIGURE 4:**
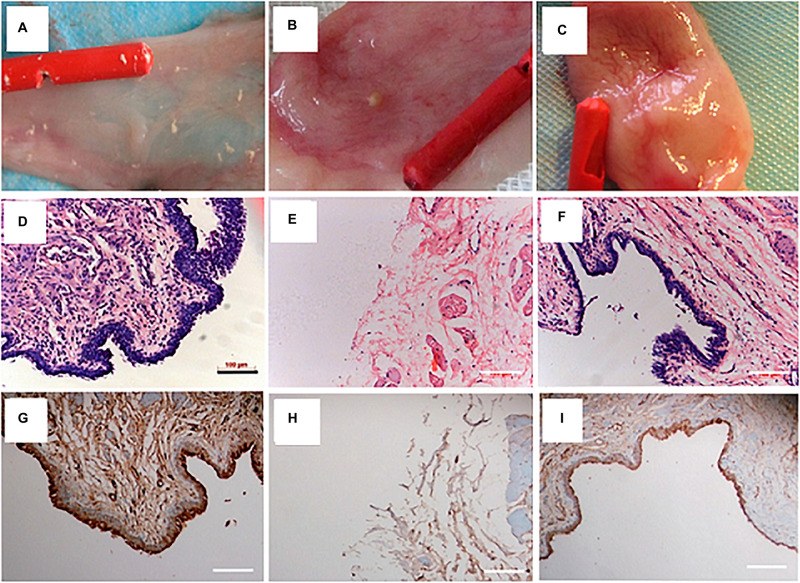
Urethral reconstruction with electrospun PLLA/PEG scaffold seeded with human amniotic mesenchymal stem cells in rabbit model. Macroscopic view of urethral mucosa regeneration in **(A)** sham operated **(B)** unseeded scaffold implanted, and **(C)** pre-seeded scaffold implanted groups after 12 weeks of follow-up. H&E staining of urethral tissue at 12 weeks after operation in **(D)** sham **(E)** unseeded, and **(F)** pre-seeded groups. Immunohistochemical staining of urethral tissues by AE1/AE3 marker at 12 weeks after operation in **(G)** sham **(H)** unseeded, and **(I)** pre-seeded groups. Reprinted from Lv et al. (2016).

The incorporation of bioactive molecules is a popular potential option during the fabrication of electrospun fibrous scaffolds. The high surface/volume ratio of electrospun scaffolds provides a great opportunity for loading and vast delivering of specified molecules during the regeneration process (Pant et al., 2019). Zhang et al. (2015) designed a collagen/PLCL electrospun scaffold loaded with Wnt pathway inhibitor (ICG-001) for urethroplasty and studied its potential for inhibiting the ECM overexpression, the often-occurring problem in urethral defect healing which reduces the urethral caliber and impairment to the flow of urine. *In vitro* results demonstrated the excellent biocompatibility of the ICG-001 and its effectiveness in inhibiting ECM expression of fibroblast. After 3 months of implantation in rabbits urethral defect, urethral strictures, and fistulas were found in those treated with non-ICG-001 delivering scaffolds, while all the rabbits treated with ICG-001-delivering scaffolds showed wide caliber in the urethras. In addition, less collagen, more smooth muscle, and thicker epithelium was observed in urethras repaired with ICG-001 delivering scaffolds. In one study, Lv et al. fabricated a composite scaffold of PLLA with PEG to increase the hydrophilicity and cell attachment properties of PLLA. PLLA/PEG scaffold was seeded with human amniotic mesenchymal cells and employed for urethral reconstruction in rabbit for a segment of 2^∗^1.5 cm. After 12 weeks of follow-up, based on retrograde urethrogram, no signs of stricture and fistula were detected in the pre-seeded group compared to unseeded and sham-operated animals. Besides, a multilayered urothelium and SMCs aligned along the scaffold fibers were present in the pre-seeded group (Lv et al., 2016; Figure 4). Taking all the above mentioned into account, it seems that the scaffold design is the central point for urethral tissue regeneration. In addition, the quality of the regeneration process can be improved through optimizing the cell seeding method and by incorporation of different bioactive molecules to the polymeric scaffold (Table 2).

## Ureter Tissue Engineering

Ureters deliver urine from renal pelvis to the urinary bladder, which mainly takes place by two factors: (i) peristaltic contractions of muscular layer in the wall of the ureter and, (ii) pressure gradient of urine (Gómez-Blanco et al., 2016). The ureter wall contains three layers of mucosa, muscularis, and adventitia (Figure 2A). The mucosa is composed of a thick epithelial lining of UCs with typical characteristics of a urothelium layer. It is highly folded, creating a characteristic star-shaped lumen (Pawlina and Ross, 2018). The muscularis at mid-ureteral wall is composed of a mostly longitudinally oriented inner layer and a circular outer layer; however, this separation is not sharp, and fascicles of both layers are oblique and blend into one another. At lower 2–3 cm of the ureter near the bladder, another outer longitudinal layer of muscle is present that is the extension of bladder detrusor attached to the adventitia. The latter muscle layer is clearly separated from ureter by a small injectable space, i.e., Waldeyer’s separation (Woodburne, 1965).

Although the ureteral injury is a relatively infrequent problem, the proper functioning of the ureters is vital for normal kidney performance. The normal function and structure of ureter can be seriously damaged in some clinical conditions. Urolithiasis, chronic inflammation, infection, and ureteral carcinoma can reason the formation of ureteral stricture (Tyritzis and Wiklund, 2015). However, iatrogenic causes account for the majority of ureteral injuries. Most of the iatrogenic cases were caused during gynecological surgeries (about 70%) (Gild et al., 2018), while other procedures in the pelvis, including colorectal, vascular pelvic, and urologic surgeries, were associated with a much lower incidence of ureter injury (Elliott and McAninch, 2006; Delacroix and Winters, 2010). The widespread application of endourological and laparoscopic surgeries during the last decades has greatly increased the incidence of iatrogenic ureteral injuries (Parpala-Sparman et al., 2008; Traxer and Thomas, 2013). Unfortunately, these injuries are less likely to be recognized during the surgery, and the delayed treatments may end in urinoma, sepsis, and even loss of renal function (Simaioforidis et al., 2013). Management of cases in which end-to-end anastomosis is not possible or not detected shortly after incidence would be an extensive and challenging procedure in the field of reconstructive urology. Different surgical modalities may be employed for the reconstruction of ureteral injuries depending on the type and location of the injury, such as ureteroneocystostomy, psoas hitch, boari flap, replacement of ureter with the ileum, transureteroureterostomy, and kidney autotransplantation (Kloskowski et al., 2013; Gild et al., 2018). However, the procedures requiring donor tissue would be complicated by shortage of donor sites; also, these modalities are followed by several complications, including excessive mucus production, chronic renal failure, infection, and formation of stricture at anastomosis site (Benson et al., 1990; Armatys et al., 2009). The complexity and high complication rate of the current reconstructive methods highlighted the need for alternative therapeutic strategies. Tissue engineering of the ureter is the most encouraging substitute approach for reconstructive goals, although the number and quality of works in this field are essentially inadequate (Figure 2D).

An ideal construct for ureter tissue engineering should be flexible and retains the luminal patency to ensure safe urine passage. In addition, it should be non-immunogenic and impermeable to urine while providing a suitable substrate for cell colonization and tissue remodeling. The complexity of surgery procedure, toxic effect of urine, and high level of minimum requirements for candidate constructs have hindered the advances in this area. Different strategies of preparation and application of tissue-engineered constructs for ureter tissue reconstruction have been studied so far (El-Hakim et al., 2005; Wolfe et al., 2011; Zhao et al., 2012, 2016; Salehipour et al., 2013; de Jonge P. K. et al., 2018; Janke et al., 2019). As urinary diversion tissue engineering holds relatively similar requirements and problems to the ureter, the reconstructive approaches are quite comparable. Since ureter injuries are usually acute and require immediate management, surgeons need a graft ready for implantation, so most of the research in this field are dedicated to design a off-the-shelf grafts. Natural scaffolds, such as SIS, amniotic membrane, and decellularized ureter, were the most used materials, which have been employed for reconstruction of the ureter in dog, pig, rabbit, and rat models (Xie et al., 2000; Osman et al., 2004; Salehipour et al., 2013). In addition, collagen sponge (Tachibana et al., 1985; de Jonge P. et al., 2018), Gore-Tex (Baltaci et al., 1998), and electrospun PLCL (Kloskowski et al., 2014) scaffold were tested, as bare scaffolds, for ureter tissue regeneration. Nevertheless, insignificant levels of regeneration were observed in all studies, and a similar array of complications was reported. Although the UCs showed high growing and covering potential, the smooth muscles were unable to immigrate into the implanted scaffold and remained limited to the anastomosis site. The outcomes of using scaffolds as off-the-shelf products were unsatisfactory, as fibrosis, graft contraction, obstruction, and hydronephrosis were frequently reported in this setting.

Pre-seeding of scaffolds is a generally accepted strategy for preventing fibrosis and graft shrinkage. The cultured cells could also improve remodeling, neovascularization, degradation rate, and immune tolerance of the engineered constructs (Muschler et al., 2004; Liao et al., 2008; Xue et al., 2016). In this regard, EL-Hakim et al. cultured autologous porcine bladder cells (UCs and SMCs) on SIS and utilized the pre-seeded scaffolds for the reconstruction of a 5-cm segment of the ureter. However, the cell seeding did not improve functional outcomes of ureter regeneration; as obstruction, graft contraction, and hydronephrosis were detected in both groups (El-Hakim et al., 2005). In another study, Zhao et al. differentiated adipose-derived stem cells into SMCs and seeded the induced differentiated cells on the vessel extracellular matrix. Reconstruction of rabbit ureter with this pre-seeded scaffold revealed acceptable outcome, and no ureteral stricture or hydroureteronephrosis was reported (Zhao et al., 2012). Therefore, the supportive role of pre-seeding of scaffolds for ureter regeneration, owing to the small number of works, has been remained unclear.

Preimplantation of scaffolds in heterotopic sites [functionally unrelated sites such as omentum (Meng et al., 2015) or subcutis (de Jonge P. K. et al., 2018)] is an alternative strategy for the preparation of tissue-engineered scaffolds for ureter construction. The main goals of this procedure are the initiation of vasculature development, and priming the remolding process in engineered constructs. This idea has been studied with both bare and pre-seeded scaffolds (Meng et al., 2015; de Jonge P. K. et al., 2018). de Jonge P. K. et al. (2018) utilized a subcutaneously preimplanted tubularized collagen-Vicryl scaffold in goat for ureter reconstruction. They reported predominantly healthy kidneys in the preimplanted group compared to the bare collagen-Vicryl scaffold group with fibrotic and inflamed regions in the kidneys; however, both groups presented incomplete muscle layer development limited to the anastomosis site. In this line, bladder submucosa and acellular matrix were seeded with stem cells, and SMCs followed by preimplantation in rabbit omentum. The functional outcome of the ureter reconstruction with these constructs was very promising as no hydronephrosis and stricture were reported (Liao et al., 2013; Meng et al., 2015). Although the preimplantation method presented the most encouraging outcomes for the ureter reconstruction, clinical translation of this approach seems to be limited by its complexity and lengthy preparation procedure. In sum, tissue engineering of ureter is in the earliest steps for discovering appropriate biomaterials and fabrication processes.

## Electrospinning for Ureteral Tissue Engineering

Scaffold plays a vital role in the regeneration of ureteral tissue and must possess several special biophysical and biochemical properties. The scaffold has to be biocompatible and provide a suitable microstructure for urothelial and SMCs colonization and growth (Shen et al., 2010; Fu et al., 2012). It should have an analogous degradation rate to its remodeling time and should be strong enough to withstand the visceral pressure and remain patent until a confluent urothelium covers its luminal surface (Wang et al., 2015a,b). Kloskowski et al. (2014) studied acellular aortic arch (AAM) and electrospun PLCL scaffolds in a rat model with the aim of comparing natural and synthetic scaffolds for ureter reconstruction (Table 2). They observed that AAM had higher wettability and porosity, which facilitated the cell growth and colonization and could easily integrate with the surrounding tissue. However, urine can permeate through AAM that is toxic to the cells and may cause tissue irritation. The PLCL scaffolds on the other hand offered a less favorable platform for cell colonization, but provided an impermeable barrier for urine, isolating the newly formed tissue from its toxic effects. In sum, based on PLCL performance in animal model, they concluded that electrospun scaffold of PLCL copolymer revealed superior properties for ureter tissue engineering than naturally derived aortic arch; however, the wettability and thickness of the scaffold should be optimized for the best performance.

Considering the fact that electrospinning enables the production of biomimetic nanofibrous structure to native ECM with controllable composition, most of the studies have designed composite electrospun structures to address the complex requirements of a ureteral scaffold (Shen et al., 2010; Fu et al., 2012; Shi et al., 2012; Wang et al., 2015a; Xu et al., 2015). Shen et al. (2010) used a blend of PCL and lecithin for the fabrication of an electrospun tubular construct. Rabbit UCs survived on this scaffold for only 7 days after subcutaneous transplantation into nude mice. Nevertheless, when UCs cultured on the pre-seeded scaffolds with rabbit bone marrow mesenchymal stem cells, they survived and proliferated for 30 days. Improved growth of UCs in the pre-seeded group appeared to be the consequence of significantly higher neovascularization, which in turn could be related to the established potential of bone marrow mesenchymal stem cells for the promotion of angiogenesis through secretion of several growth factors including basic fibroblast growth factor, vascular endothelial growth factor, and angiopoietin (Shen et al., 2010). In this regard, an electrospun composite scaffold of PLLA-collagen, configured as a ureteral graft, demonstrated suitable supportive characteristics for the culture of UCs (Fu et al., 2012; Shi et al., 2012). Xu et al. fabricated a tissue-specific scaffold by electrospinning a mixture of PLLA and ureteral extracellular matrix (UECM) as a mechanical reinforcer and biocompatibility enhancer, respectively (Xu et al., 2015). They evaluated the phenotype of UCs on the scaffolds and reported a significantly superior proliferation on PLLA/UECM scaffold as compared to pure PLLA or PLLA-small intestinal submucosa (PLLA-SIS) scaffolds. In addition, the expression of a UC-specific marker was significantly higher in the tissue-specific scaffolds than in the pure PLLA or PLLA-SIS scaffolds at different time points. They concluded that the favorable outcomes of PLLA-UECM could be attributed to its similarity with the *in vivo* microenvironment. In order to eliminate the chance of ureteral obstruction caused by degrading stent fragments in the distal ureter, which may lead to hydronephrosis and renal damages, Wang et al. (2015a) employed a double-needle electrospinning method to fabricate a ureteral stent with gradient degradation rate from distal to the proximal end. They produced a tubular construct containing gradient components of PCL and PLGA by loading different PCL/PLGA solutions (15–85 and 25–75%, PCL and PLGA, respectively) into two separate syringes along a rotating mandrel. Due to the higher PLGA content at the distal end, the stents were gradually degraded from the distal to the proximal end in a porcine model with no ureteral obstruction. In addition, the PCL/PLGA stent exhibited more biocompatible features than the Shagong^®^, a standard biostable stent, considering foreign-body reactions, tissue inflammation, and edema. Although a broad range of biomaterials can be studied for the ureter reconstruction by electrospun seamless tubular scaffolds, few works have been dealt with this issue. It seems that complex surgical procedures for implantation of the ureter constructs and the essential need for big animal models imposed the main limitations in the development of this field.

## Conclusion

Although pioneer works at the beginning of the third millenium opened very exciting vistas for reconstructive urology through tissue engineering strategies, these achievements were not followed and improved by future studies. Therefore, new biomaterials and novel innovative fabrication methods (such as 3D bioprinting, combination of current fabrication methods, etc.) for tissue engineering of urinary tract organs are still welcomed. Control and optimization of cells’ function and delivery of bioactive molecules are issues that were neglected in primary works. The electrospinning procedure by providing a relatively simple and versatile method for the fabrication of nanofibers has provided an interesting platform for addressing these issues.

Resilient and load bearing nature of bladder mandate the integration of electrospinning procedure with other fabrication methods to construct hybrid scaffolds capable of withstanding the cyclic mechanical loads while providing the ECM mimetic nanostructure. These types of constructs will possess suitable mechanical features, cell-controlling cues, and favorable nano-sized urine interface in the same context.

The urethra, on the other hand, has simpler structure and functions and its regeneration majorly relies on cellular interaction. Therefore, electrospun scaffolds can offer very encouraging substrates for concurrent implantation of cells and delivery of bioactive agents that conduct the proper regeneration by inhibition of the cells that participate in the formation of strictures. Application of electrospun scaffolds for urethra tissue engineering stands in its very beginning steps of development, and numerous regenerative schemes in this area can be designed.

Among the three organs reviewed in this article, the ureter is the least studied one due to the relatively few cases requiring ureter reconstruction. However, the challenging nature of ureter reconstruction justifies reconstructive approaches via tissue engineering methods. The electrospinning technique has easily adaptable configures for the fabrication of tubular constructs, and different biomaterials can be integrated through this method for the production of composite constructs that are more compatible with the natural structure of the ureter.

Taken together, the electrospinning method offers very interesting and diverse options for researchers who are investigating tissue engineering of urinary tract organs. This method provides appropriate constructs for studying cells interaction, biomaterials integration, innovative molding, and functional evaluation.

## Author Contributions

MZ and MN wrote the manuscript. NS and SR reviewed the article before publication. All authors contributed to the article and approved the submitted version.

## Conflict of Interest

The authors declare that the research was conducted in the absence of any commercial or financial relationships that could be construed as a potential conflict of interest.
