# Supplementary material for: Electrospinning: Application and Prospects for Urologic Tissue Engineering
Source: Front Bioeng Biotechnol. 2020 Oct 7;8:579925. doi: 10.3389/fbioe.2020.579925 (PMC7576678; doi:10.3389/fbioe.2020.579925)
Supplement: Supplementary file 1 [file Data_Sheet_1.docx]

**Search Component-1-1: Bladder**

urinary bladder[MeSH Terms] OR urinary bladder[tiab] OR (urinary[tiab] AND bladder[tiab]) OR bladder[tiab] OR bladder detrusor muscle[tiab] OR cystostomy[MeSH Terms] OR cystostomy[tiab] OR Cystostomies[tiab] OR Urinary Catheterization[MeSH Terms] OR Urinary Catheterization[tiab] OR (Urinary[tiab] AND Catheterization[tiab]) OR Urinary Catheterizations[tiab] OR (urinary[tiab] AND catheterizations[tiab]) OR urothelium[MeSH Terms] OR urothelium[tiab] OR urinary tract epithelium[tiab] OR (urinary tract[tiab] AND epithelium[tiab]) OR bladder tissue[tiab] OR (bladder[tiab] AND tissue[tiab]) OR urinary bladder tissue[tiab] OR (urinary bladder[tiab] AND tissue[tiab]) OR bladder augmentation[tiab] OR (bladder[tiab] AND augmentation[tiab]) OR urinary bladder augmentation[tiab] OR (urinary bladder[tiab] AND augmentation[tiab]) OR bladder compliance[tiab] OR (bladder[tiab] AND compliance[tiab]) OR urinary bladder compliance[tiab] OR (urinary bladder[tiab] AND compliance[tiab]) OR bladder capacity[tiab] OR (bladder[tiab] AND capacity[tiab]) OR urinary bladder capacity[tiab] OR (urinary bladder[tiab] AND capacity[tiab]) OR bladder wall[tiab] OR (bladder[tiab] AND wall[tiab]) OR urinary bladder wall[tiab] OR (Urinary bladder[tiab] AND wall[tiab]) OR cystectomy[MeSH Terms] OR cystectomy[tiab] OR cystoplasty[tiab]

**Search Component-1-2: Urethra**

urethra[MeSH Terms] OR urethra[tiab] OR urethras[tiab] OR urethral stricture[MeSH Terms] OR urethral stricture[tiab] OR (urethral[tiab] AND stricture[tiab]) OR urethral strictures[tiab] OR (urethral[tiab] AND strictures[tiab]) OR urethral stenosis[tiab] OR (urethral[tiab] AND stenosis[tiab]) OR anterior urethral strictures[tiab] OR (anterior[tiab] AND urethral strictures[tiab]) OR anterior urethral stricture[tiab] OR (anterior[tiab] AND urethral stricture[tiab]) OR posterior urethral stricture[tiab] OR (posterior[tiab] AND urethral stricture[tiab]) OR posterior urethral strictures[tiab] OR (posterior[tiab] AND urethral strictures[tiab]) OR urinary catheterization[MeSH Terms] OR urinary catheterization[tiab] OR (urinary[tiab] AND catheterization[tiab]) OR urinary catheterizations[tiab] OR (urinary[tiab] AND catheterizations[tiab]) OR urethral catheterizations[tiab] OR (urethral[tiab] AND catheterizations[tiab]) OR urethral catheterization[tiab] OR (urethral[tiab] AND catheterization[tiab]) OR foley catheterization[tiab] OR (foley[tiab] AND catheterization[tiab]) OR intermittent urethral catheterization[tiab] OR urethral stent[tiab] OR (urethral[tiab] AND stent[tiab]) OR urethral stents[tiab] OR (urethral[tiab] AND stents[tiab]) OR urethral obstruction[MeSH Terms] OR urethral obstruction[tiab] OR (urethral[tiab] AND obstruction[tiab]) OR urethral obstructions[tiab] OR (urethral[tiab] AND obstructions[tiab]) OR meatal stenosis[tiab] OR (meatal[tiab] AND stenosis[tiab]) OR Urethroplasty

**Search Component-1-3: Ureter**

Ureter[MeSH Terms] OR ureter[tiab] OR ureters[tiab] OR ureterostomy[MeSH Terms] OR ureterostomy[tiab] OR ureterostomies[tiab] OR urinary diversion[MeSH Terms] OR urinary diversion[tiab] OR (urinary[tiab] AND diversion[tiab]) OR urinary diversions[tiab] OR (urinary[tiab] AND diversions[tiab]) OR ileal conduit[tiab] OR (ileal[tiab] AND conduit[tiab]) OR ileal conduits[tiab] OR (ileal[tiab] AND conduits[tiab]) OR ureteral[tiab] OR ureterotomy[tiab] OR ureterotomies[tiab]

**Search Component-2: Tissue engineering and Scaffolding**

tissue engineering[MeSH Terms] OR tissue engineering[tiab] OR (tissue[tiab] AND engineering[tiab]) OR organ culture techniques[MeSH Terms] OR organ culture techniques[tiab] OR (organ culture[tiab] AND techniques[tiab]) OR organ culture technique[tiab] OR (organ culture[tiab] AND technique[tiab]) OR organ culture[tiab] OR (organ[tiab] AND culture[tiab]) OR culture technique[tiab] OR (culture[tiab] AND technique[tiab]) OR culture techniques[tiab] OR (culture[tiab] AND techniques[tiab]) OR organoids[MeSH Terms] OR organoids[tiab] OR organoid[tiab] OR guided tissue regeneration[MeSH Terms] OR guided tissue regeneration[tiab] OR (guided[tiab] AND tissue egeneration[tiab]) OR tissue regeneration[tiab] OR (tissue[tiab] AND regeneration[tiab]) OR regeneration[MeSH Terms] OR regeneration[tiab] OR bioengineering[MeSH Terms] OR bioengineering[tiab] OR Bio-Engineering[tiab] OR biological engineering[tiab] OR (biological[tiab] AND engineering[tiab]) OR tissue scaffolds[MeSH Terms] OR tissue scaffolds[tiab] OR (tissue[tiab] AND scaffolds[tiab]) OR tissue scaffold[tiab] OR (tissue[tiab] AND scaffold[tiab]) OR tissue scaffolding[tiab] OR (tissue[tiab] AND scaffolding[tiab]) OR tissue scaffoldings[tiab] OR (tissue[tiab] AND scaffoldings[tiab]) OR matrix[tiab] OR matrices[tiab] OR biomatrix[tiab] OR biomatrices[tiab] OR extracellular matrix[MeSH Terms] OR extracellular matrix[tiab] OR (extracellular[tiab] AND matrix[tiab]) OR ECM[tiab] OR biocompatible materials[MeSH Terms] OR biocompatible materials[tiab] OR (biocompatible[tiab] AND materials[tiab]) OR biocompatible material[tiab] OR (biocompatible[tiab] AND material[tiab]) OR biomaterials[tiab] OR biomaterial[tiab] OR bioartificial materials[tiab] OR (bioartificial[tiab] AND materials[tiab]) OR bioartificial material[tiab] OR (bioartificial[tiab] AND material[tiab]) OR transplants[MeSH Terms] OR transplants[tiab] OR transplant[tiab] OR graft[tiab] OR grafts[tiab] OR tissue transplants[tiab] OR (tissue[tiab] AND transplants[tiab]) OR tissue transplant[tiab] OR (tissue[tiab] AND transplant[tiab]) OR tissue grafts[tiab] OR (tissue[tiab] AND grafts[tiab]) OR tissue graft[tiab] OR (tissue[tiab] AND graft[tiab]) OR organ transplants[tiab] OR (organ[tiab] AND transplants[tiab]) OR organ transplant[tiab] OR (organ[tiab] AND transplant[tiab]) OR organ grafts[tiab] OR (organ[tiab] AND grafts[tiab]) OR organ graft[tiab] OR (organ[tiab] AND graft[tiab]) OR autografts[MeSH Terms] OR autografts[tiab] OR autograft[tiab] OR autologous transplants[tiab] OR (autologous[tiab] AND transplants[tiab]) OR autologous transplant[tiab] OR (autologous[tiab] AND transplant[tiab]) OR autotransplants[tiab] OR autotransplant[tiab] OR biomimetic materials[MeSH Terms] OR biomimetic materials[tiab] OR (biomimetic[tiab] AND materials[tiab) OR biomimetic material[tiab] OR (biomimetic[tiab] AND material[tiab]) OR biomimicry materials[tiab] OR (biomimicry[tiab] AND materials[tiab]) OR biomimicry material[tiab] OR (biomimicry[tiab] AND material[tiab]) OR artificial organs[MeSH Terms] OR artificial organs[tiab] OR (artificial[tiab] AND organs[tiab]) OR artificial organ[tiab] OR (artificial[tiab] AND organ[tiab]) OR bio-artificial[tiab] OR bioartificial[tiab] OR bioartificial organs[MeSH Terms] OR bioartificial organs[tiab] OR (bioartificial[tiab] AND organs[tiab]) OR bioartificial organ[tiab] OR (bioartificial[tiab] AND organ[tiab]) OR biomimetics[MeSH Terms] OR biomimetics[tiab] OR biomimetic[tiab] OR bio-inspired engineering[tiab] OR (bio-inspired[tiab] AND engineering[tiab]) OR bioinspired engineering[tiab] OR (bioinspired[tiab] AND engineering[tiab]) OR biomimicry engineering[tiab] OR (biomimicry[tiab] AND engineering[tiab]) OR biological mimetic[tiab] OR (biological[tiab] AND mimetic[tiab]) OR biological mimetics[tiab] OR (biological[tiab] AND mimetics[tiab]) OR regenerative medicine[MeSH Terms] OR regenerative medicine[tiab] OR (regenerative[tiab] AND medicine[tiab]) OR reconstruction[tiab] OR reconstructed[tiab] OR reconstructing[tiab] OR tissue engineered[tiab] OR (tissue[tiab] AND engineered[tiab]) OR bioengineered substitutes[tiab] OR (bioengineered[tiab] AND substitutes[tiab]) OR bioengineered substitute[tiab] OR (bioengineered[tiab] AND substitute[tiab]) OR cell-free scaffold[tiab] OR cell-free scaffolds[tiab] OR bare scaffold[tiab] OR bare scaffolds[tiab] OR cell-seeded scaffold[tiab] OR (cell-seeded[tiab] AND scaffold[tiab]) OR cell-seeded scaffolds[tiab] OR (cell-seeded[tiab] AND scaffolds[tiab]) OR cell-seeded matrix[tiab] OR (cell-seeded[tiab] AND matrix[tiab]) OR cell-seeded matrices[tiab] OR (cell-seeded[tiab] AND matrices[tiab]) OR hybrid scaffold[tiab] OR hybrid scaffolds[tiab] OR hybrid matrix[tiab] OR hybrid matrices[tiab] OR composite scaffold[tiab] OR composite scaffolds[tiab] OR (composite[tiab] AND scaffolds[tiab]) OR (composite[tiab] AND scaffold[tiab]) OR composite matrix[tiab] OR composite matrices

**Search Component-3: Electrospinning**

electrospinning[tiab] OR electrospinning process[tiab] OR (electrospinning[tiab] AND process[tiab]) OR electrospinning procedure[tiab] OR (electrospinning[tiab] AND procedure[tiab]) OR electrospinning method[tiab] OR (electrospinning[tiab] AND method[tiab]) OR electrospinning fabrication[tiab] OR (electrospinning[tiab] AND fabrication[tiab]) OR electrospinning nanofiber[tiab] OR (electrospinning[tiab] AND nanofiber[tiab]) OR electrospun[tiab] OR electro-spun[tiab] OR electrospun mat[tiab] OR (electrospun[tiab] AND mat[tiab]) OR electrospun mats[tiab] OR (electrospun[tiab] AND mats[tiab]) OR electrospun nanofiber[tiab] OR (electrospun[tiab] AND nanofiber[tiab]) OR electrospun nanofibers[tiab] OR (electrospun[tiab] AND nanofibers[tiab]) OR electrospun membrane[tiab] OR (electrospun[tiab] AND membrane[tiab]) OR electrospun membranes[tiab] OR (electrospun[tiab] AND membranes[tiab]) OR electrospun construct[tiab] OR (electrospun[tiab] AND construct[tiab]) OR electrospun constructs[tiab] OR (electrospun[tiab] AND constructs[tiab]) OR electrospun scaffold[tiab] OR (electrospun[tiab] AND scaffold[tiab]) OR electrospun scaffolds[tiab] OR (electrospun[tiab] AND scaffolds[tiab]) OR electrospun structure[tiab] OR (electrospun[tiab] AND structure[tiab]) OR electrospun structures[tiab] OR (electrospun[tiab] AND structures[tiab]) OR nanostructures[MeSH Terms] OR nanostructures[tiab] OR nanostructure[tiab] OR nanostructured materials[tiab] OR (nanostructured[tiab] AND materials[tiab]) OR nanostructured material[tiab] OR (nanostructured[tiab] AND material[tiab]) OR nanomaterials[tiab] OR nanomaterial[tiab] OR nanofibers[MeSH Terms] OR nanofibers[tiab] OR nanofiber[tiab] OR nano-scaffold[tiab] OR nanoscaffold[tiab] OR nano-matrix[tiab] OR nanomatrix[tiab] OR nano-scale[tiab] OR nanoscale [tiab] OR nano[tiab]

- Search Component-1-1 **AND** Search Component-2 **AND** Search Component-3: Supplementary Figure 1
- Search Component-1-2 **AND** Search Component-2 **AND** Search Component-3: Supplementary Figure 2
- Search Component-1-3 **AND** Search Component-2 **AND** Search Component-3: Supplementary Figure 3


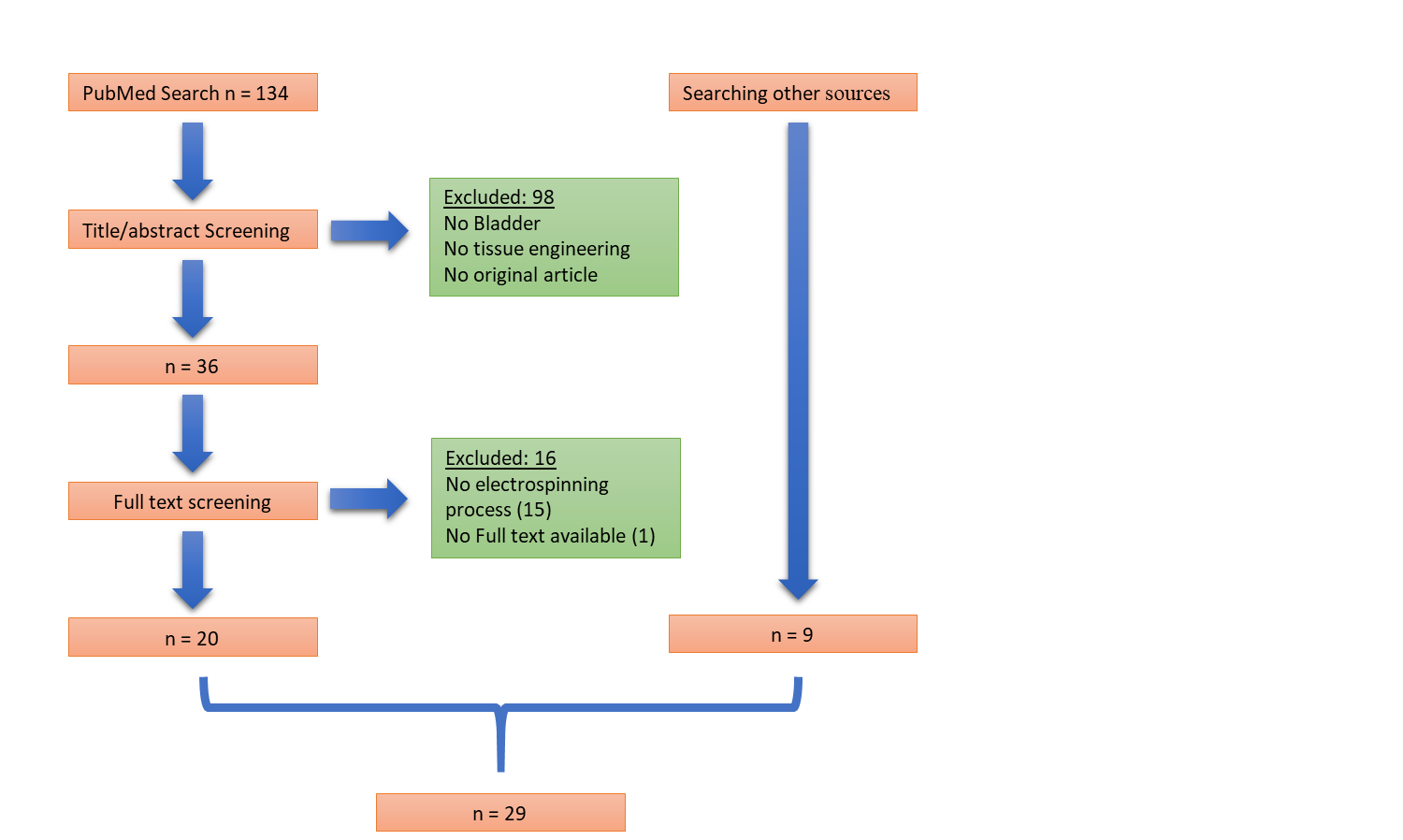


Supplementary Figure 1. Flowchart of search and screening procedure for studies concerning bladder tissue engineering using the electrospinning method (5^th^ of May 2019).


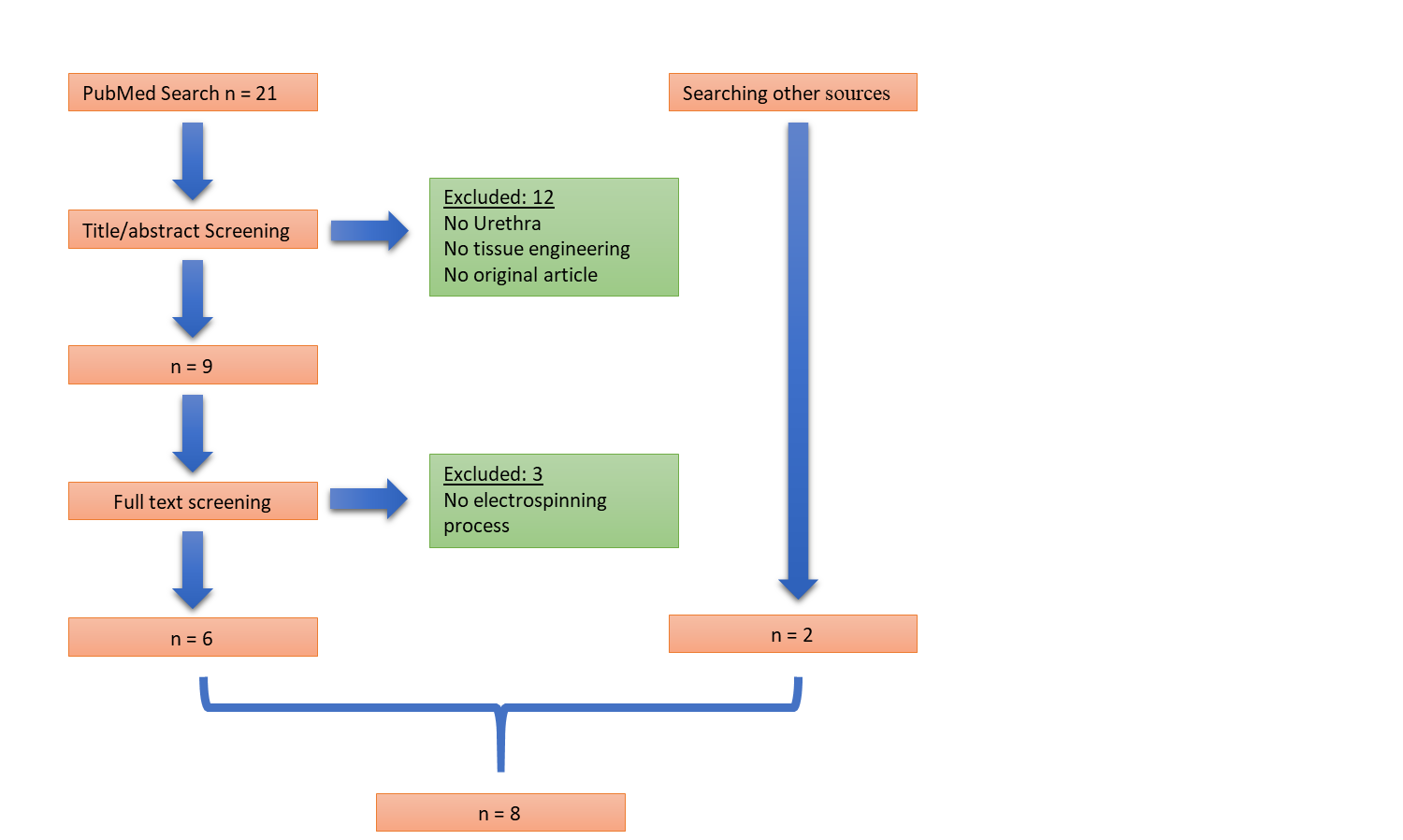


Supplementary Figure 2. Flowchart of search and screening procedure for studies concerning urethra tissue engineering using the electrospinning method (5^th^ of May 2019).


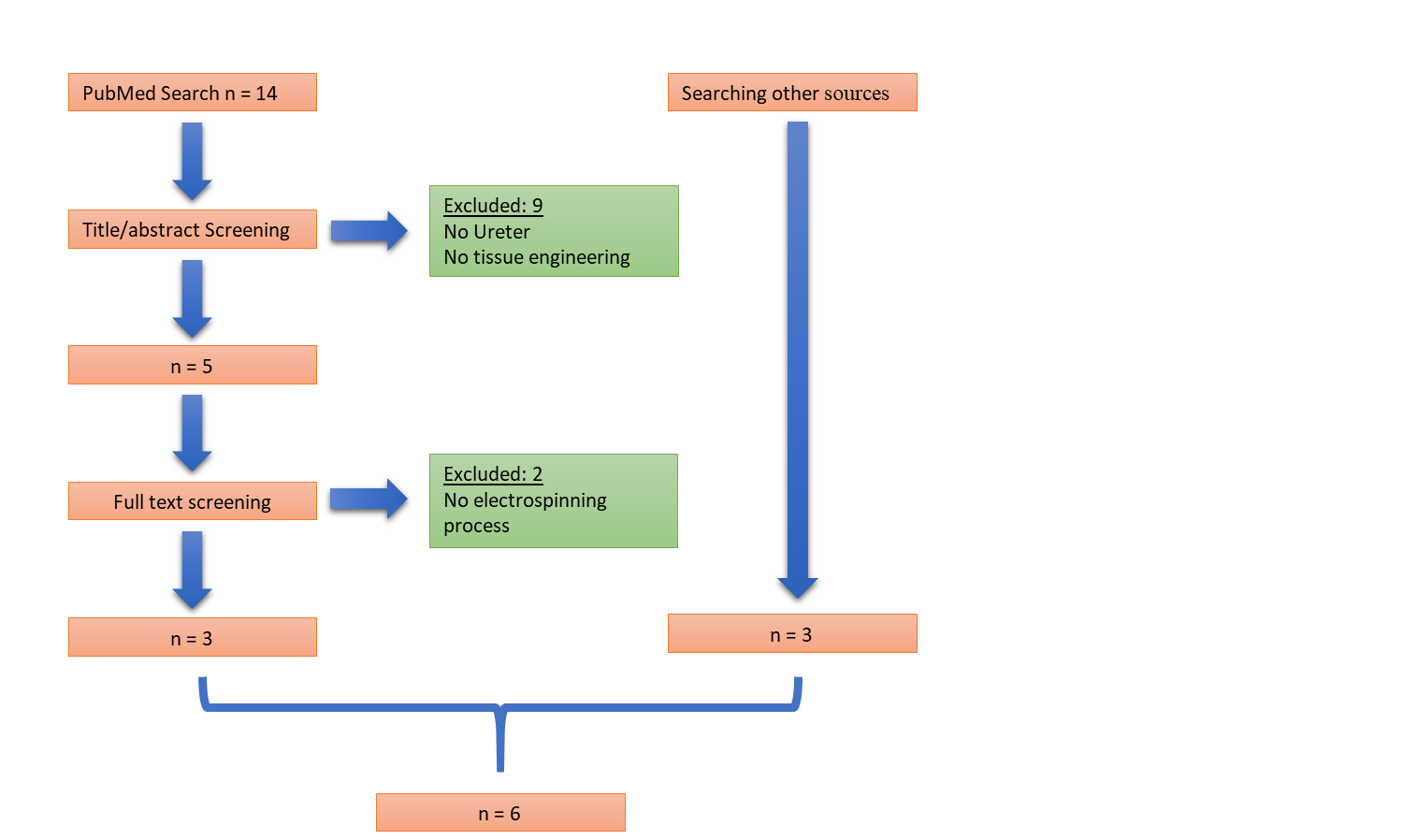


Supplementary Figure 3. Flowchart of search and screening procedure for studies concerning ureter tissue engineering using the electrospinning method (5^th^ of May 2019).
